# Characterization of mitochondrial DNA quantity and quality in the human aged and Alzheimer’s disease brain

**DOI:** 10.1186/s13024-021-00495-8

**Published:** 2021-11-06

**Authors:** Hans-Ulrich Klein, Caroline Trumpff, Hyun-Sik Yang, Annie J. Lee, Martin Picard, David A. Bennett, Philip L. De Jager

**Affiliations:** 1 Center for Translational & Computational Neuroimmunology, Department of Neurology, Columbia University Irving Medical Center, New York, NY 10032 USA; 2 Taub Institute for Research on Alzheimer’s Disease and the Aging Brain, Columbia University Irving Medical Center, New York, NY 10032 USA; 3 Division of Behavioral Medicine, Department of Psychiatry, Columbia University Irving Medical Center, New York, NY 10032 USA; 4 Center for Alzheimer Research and Treatment, Department of Neurology, Brigham and Women’s Hospital, Boston, MA 02115 USA; 5 Merritt Center and Columbia Translational Neuroscience Initiative, Department of Neurology, Columbia University Irving Medical Center, New York, NY 10032 USA; 6 Rush Alzheimer’s Disease Center, Rush University Medical Center, Chicago, IL 60612 USA

**Keywords:** Mitochondria, Neurodegeneration, Alzheimer’s disease, Mitochondrial DNA copy number, Mitochondrial heteroplasmy, Tau, Amyloid, TDP-43

## Abstract

**Background:**

Mitochondrial dysfunction is a feature of neurodegenerative diseases, including Alzheimer’s disease (AD). Changes in the mitochondrial DNA copy number (mtDNAcn) and increased mitochondrial DNA mutation burden have both been associated with neurodegenerative diseases and cognitive decline. This study aims to systematically identify which common brain pathologies in the aged human brain are associated with mitochondrial recalibrations and to disentangle the relationship between these pathologies, mtDNAcn, mtDNA heteroplasmy, aging, neuronal loss, and cognitive function.

**Methods:**

Whole-genome sequencing data from *n* = 1361 human brain samples from 5 different regions were used to quantify mtDNAcn as well as heteroplasmic mtDNA point mutations and small indels. Brain samples were assessed for 10 common pathologies. Annual cognitive test results were used to assess cognitive function proximal to death. For a subset of samples, neuronal proportions were estimated from RNA-seq profiles, and mass spectrometry was used to quantify the mitochondrial protein content of the tissue.

**Results:**

mtDNAcn was 7–14% lower in AD relative to control participants. When accounting for all 10 common neuropathologies, only tau was significantly associated with lower mtDNAcn in the dorsolateral prefrontal cortex. In the posterior cingulate cortex, TDP-43 pathology demonstrated a distinct association with mtDNAcn. No changes were observed in the cerebellum, which is affected late by pathologies. Neither age nor gender was associated with mtDNAcn in the studied brain regions when adjusting for pathologies. Mitochondrial content and mtDNAcn independently explained variance in cognitive function unaccounted by pathologies, implicating complex mitochondrial recalibrations in cognitive decline. In contrast, mtDNA heteroplasmy levels increased by 1.5% per year of life in the cortical regions, but displayed no association with any of the pathologies or cognitive function.

**Conclusions:**

We studied mtDNA quantity and quality in relation to mixed pathologies of aging and showed that tau and not amyloid-β is primarily associated with reduced mtDNAcn. In the posterior cingulate cortex, the association of TDP-43 with low mtDNAcn points to a vulnerability of this region in limbic-predominant age-related TDP-43 encephalopathy. While we found low mtDNAcn in brain regions affected by pathologies, the absence of associations with mtDNA heteroplasmy burden indicates that mtDNA point mutations and small indels are unlikely to be involved in the pathogenesis of late-onset neurodegenerative diseases.

**Supplementary Information:**

The online version contains supplementary material available at 10.1186/s13024-021-00495-8.

## Background

Mitochondria are complex multi-functional organelles involved in various pathways including fatty acid and cholesterol synthesis, apoptosis, calcium signaling, and adenosine triphosphate generation [1, 2]. Dysfunctional mitochondria have been described in aging [3] and in many neurodegenerative diseases such as Alzheimer’s disease (AD) and amyotrophic lateral sclerosis (ALS) [4]. Mitochondria harbor their own circular genome of 16,569 base pairs encoding 13 proteins of the respiratory chain. Mitochondrial DNA (mtDNA) can be replicated independent of the cell cycle. Since mtDNA expression is required for respiratory activity, the mtDNA copy number (mtDNAcn) within a cell is regulated to meet the cell’s metabolic needs, resulting in a wide range of mtDNAcn in different tissues and conditions [5, 6]. Like nuclear DNA, mtDNA can also carry mutations, which either affect all copies of the mtDNA in a cell (termed homoplasmy) or only a fraction of the mtDNA molecules (termed heteroplasmy). Heteroplasmic mutations are assumed to be somatically generated or inherited as low level variants, and they can clonally expand over an individual’s life-time [7].

The mtDNAcn has become a popular potential marker of mitochondrial health in translational studies, because mtDNAcn can be measured in stored biospecimens at large scale using qPCR or DNA sequencing techniques. In AD, several studies have investigated mtDNAcn in tissue homogenates from different brain regions and found either a lower mtDNAcn in AD or no significant changes. One of the strongest reductions (50%) was reported by an early study of the frontal cortex [8]. Smaller effect sizes or non-significant changes were reported for the hippocampus, cerebellar cortex, and cerebellum, indicating the possibility of brain region-specific effects [9–11]. Similar results were reported for other neurodegenerative diseases [5]. Interestingly, although mtDNAcn derived from whole blood is often confounded by variation in cell type composition between individuals, a recent study found an association with AD suggesting that the mtDNAcn in whole blood could potentially reflect metabolic health across tissues [12]. While the mtDNAcn overall seems to be reduced in brain regions affected by neurodegenerative diseases, mixed results have been reported for the effect of aging on mtDNAcn. For example, two studies found no evidence for age-related changes of mtDNAcn in three brain regions, skeletal muscle, and heart muscle [13, 14], whereas a more recent study reported a decrease in skeletal muscle and an increase in liver tissue with age [15].

A higher burden of mtDNA heteroplasmy has been observed in tissues from aged individuals [16]. Increased levels of heteroplasmic mtDNA deletions as well as heteroplasmic point mutations were also described in brains from AD and Parkinson’s disease (PD) patients [8, 17, 18], which lead to the hypothesis that pathogenic mtDNA mutations, when they exceed certain thresholds, could contribute to the mitochondrial dysfunction observed in late-onset neurodegenerative diseases. However, a recent high-throughput sequencing study looking at heteroplasmic point mutations found no evidence for an association with AD or PD [9].

In this study, we profiled mtDNAcn and mtDNA heteroplasmy levels in *n* = 762 brain samples from the Religious Orders Study and the Rush Memory and Aging Project (ROSMAP) [19] and implemented substantial improvements compared to previous studies: (i) The detailed pathologic characterization of ROSMAP samples facilitated the disentanglement of the effects of different brain pathologies and aging on mtDNA. Mixed pathologies are common in aged individuals including AD patients [20] and unaccounted pathologies may have contributed to some of the mixed results in the literature. (ii) Standardized cognitive tests conducted proximate to death were employed to assess the association between cognitive functioning and mtDNAcn adjusted for pathologies. (iii) Using RNA-seq-derived estimations of cell type proportions, we accounted for neuronal loss as a major confounder of mtDNAcn analyses in AD brains. (iv) We profiled five different brain regions to assess brain-regional differences (three regions in ROSMAP, and two additional regions in two independent datasets with a total of *n* = 599 additional samples). (v) Finally, we calculated a proteomic score representing mitochondrial content to investigate whether changes in mtDNAcn reflect changes of mitochondrial mass or whether they are specific to mtDNA maintenance.

## Methods

### ROSMAP cohort and neuropathologic characterization

The Religious Orders Study (ROS) and the Rush Memory and Aging Project (MAP) are two cohort studies of aging and dementia conducted by the same team of investigators and share a large common core of harmonized clinical and post-mortem data collection which allows for joint analyses [19]. Participants entered the studies without known dementia and agreed to annual clinical and cognitive assessments as well as brain donation after death.

To obtain a pathologic diagnosis of AD, a modified Bielschowsky silver stain was used to visualize neuritic plaques, diffuse plaques, and neurofibrillary tangles in five cortical regions (hippocampus, entorhinal, midfrontal, middle temporal, and inferior parietal). A board-certified neuropathologist, blinded to clinical data, determined the pathologic diagnosis of AD based on an intermediate to high likelihood of AD according to the NIA Reagan criteria. A quantitative global AD pathology score was derived from the Bielschowsky silver stains by counting and standardizing each of the pathologies neuritic plaques, diffuse plaques, and neurofibrillary tangles in each of the five cortical regions, and then averaging the standardized measures across regions. The average of these three pathology measures was then used as single score of AD pathology burden. Amyloid-β and tau tangles were assessed in 8 brain regions (hippocampus, entorhinal cortex, midfrontal cortex, inferior temporal gyrus, angular gyrus, calcarine cortex, anterior cingulate cortex, and superior frontal cortex) using immunochemistry [21, 22]. Paraffin-embedded sections were immunostained for amyloid-β using 1 of 3 monoclonal anti-human antibodies: 4G8 (1:9000; Covance Labs, Madison, WI), 6F/3D (1:50; Dako North America Inc., Carpinteria, CA), and 10D5 (1:600; Elan Pharmaceuticals, San Francisco, CA). Paired helical filament (PHF) tau tangles were labeled with an antibody specific to phosphorylated tau (AT8, Thermoscientific, Waltham, MA, USA). A computerized sampling procedure combined with image analysis software was used to calculate the percentage area occupied with amyloid-β and the density of PHFtau tangles. Composite scores were computed for overall amyloid-β burden and tau tangle density by averaging the scores obtained from the eight brain regions. The three quantitative measurements (global AD pathology, amyloid-β, and tau) were square root transformed for better statistical properties.

Presence of TDP-43 cytoplasmic inclusions in neurons and glia were determined in eight regions (amygdala, entorhinal cortex, hippocampus CA1, hippocampus dentate gyrus, anterior temporal pole cortex, midtemporal cortex, orbital frontal cortex, and midfrontal cortex) using a phosphorylated monoclonal TAR5P-1D3 (pS409/410; 1:100, Ascenion, Munich, Germany) TDP-43 antibody. Based on the absence or presence of TDP-43 pathology in the eight regions, four stages of TDP-43 distribution were recognized: none, amygdala, amygdala + limbic, amygdala + limbic + neocortical [23]. Lewy body disease was assessed in four stages (not present, nigral-predominant, limbic-type, neocortical-type). Seven regions (substantia nigra, anterior cingulate cortex, entorhinal cortex, amygdala, midfrontal cortex, superior or middle temporal cortex, inferior parietal cortex) were assessed for Lewy bodies using α-synuclein immunostaining (LB509; 1:150 or 1:100, Zymed Labs, Invitrogen, Carlsbad, CA, USA; and pSyn#64; 1:20,000; Wako Chemicals, Richmond, VA,USA) [24]. Cerebral amyloid angiopathy pathology was assessed in four neocortical regions (midfrontal, midtemporal, parietal, and calcarine cortices) using using 1 of 3 monoclonal anti-human antibodies: 4G8 (1:9000; Covance Labs, Madison, WI), 6F/3D (1:50; Dako North America Inc., Carpinteria, CA), and 10D5 (1:600; Elan Pharmaceuticals, San Francisco, CA). Similar to the protocol by Love, Chalmers [25], meningeal and parenchymal vessels were assessed for amyloid-β deposition in each region and scored from 0 to 4. Scores were averaged across the four regions and categorized into none, mild, moderate, or severe [26]. Large vessel cerebral atherosclerosis rating was made by visual inspection after paraformaldehyde fixation, at the Circle of Willis at the base of the brain, and included evaluation of the vertebral, basilar, posterior cerebral, middle cerebral, and anterior cerebral arteries and their proximal branches. Severity was graded (none or possible, mild, moderate, severe) by visual examination of the extent of involvement of each artery and number of arteries involved [27]. Arteriolosclerosis was graded by evaluating the vessels of the anterior basal ganglia for histological changes as previously described [28]. Four stages (none, mild, moderate, and severe) were recognized. The presence of one or more gross chronic cerebral infarctions was determined during gross examination and confirmed histologically. The presence of one or more chronic microinfarcts was determined on sections of a minimum of nine regions stained with hematoxylin and eosin (H&E) [29]. The presence of hippocampal sclerosis was identified by severe neuronal loss and gliosis on H&E-stained sections in CA1 or subiculum [30].

DNA for whole-genome sequencing (WGS) was extracted from the dorsolateral prefrontal cortex (DLPFC) using Qiagen’s QIAamp DNA kit (*n* = 367) or Qiagen’s AllPrep Universal kit (*n* = 87), from the posterior cingulate cortex (PCC) using Qiagen’s AllPrep Universal kit (*n* = 66), or from the cerebellum (CB) using Qiagen’s Gentra Puregene Tissue kit (*n* = 242). Sample characteristics are summarized in Table 1.

**Table 1:** Characteristics of the ROSMAP cohort

	DLPFC (*n* = 454)	PCC (n = 66)	CB (n = 242)
Sex (male)	158 (34.8%)	21 (31.8%)	71 (29.3%)
Age (years)	89.3 (6.6)	89.1 (5.6)	88 (6.7)
Pathologic AD	308 (67.8%)	45 (68.2%)	141 (58.3%)
Amyloid (% area affected)	4.6 (4.4)	3.9 (3.8)	4 (4.3)
Tau (% area affected)	7.4 (8.1)	6.8 (6.8)	6.2 (7.9)
TDP-43 (none, amygdala, limbic, neocortical)	205, 76, 97, 44 (48.6, 18.0, 23.0, 10.4%)	23, 16, 12, 12 (36.5, 25.4, 19.0, 19.0%)	96, 45, 38, 33 (45.3, 21.2, 17.9, 15.6%)
Lewy bodies (none, nigral, limbic, neocortical)	337, 7, 32, 60 (77.3, 1.6, 7.3, 13.8%)	48, 2, 2, 9 (78.7, 3.3, 3.3, 14.8%)	184, 6, 21, 25 (78.0, 2.5, 8.9, 10.6%)
Cerebral amyloid angiopathy (none, mild, moderate, severe)	92, 191, 110, 52 (20.7, 42.9, 24.7, 11.7%)	13, 25, 16, 11 (20.0, 38.5, 24.6, 16.9%)	58, 101, 51, 24 (24.8, 43.2, 21.8, 10.3%)
Cerebral atherosclerosis (none, mild, moderate, severe)	84, 212, 123, 33 (18.6, 46.9, 27.2, 7.3%)	11, 25, 19, 11 (16.7, 37.9, 28.8, 16.7%)	39, 114, 72, 15 (16.2, 47.5, 30.0, 6.2%)
Arteriolosclerosis (none, mild, moderate, severe)	136, 157, 118, 42 (30.0, 34.7, 26.0, 9.3%)	9, 26, 22, 9 (13.6, 39.4, 33.3, 13.6%)	78, 79, 65, 17 (32.6, 33.1, 27.2, 7.1%)
Gross chronic infarcts (one or more)	171 (37.7%)	22 (33.3%)	83 (34.3%)
Chronic microinfarcts (one or more)	132 (29.1%)	18 (27.3%)	70 (28.9%)
Hippocampal sclerosis (present)	39 (8.7%)	6 (9.2%)	22 (9.2%)
Post mortem interval (hours)	8.4 (5.7)	6.6 (4.7)	8.3 (6.7)

Column headers denote the brain regions where specimens for WGS were sampled. Reported pathology burdens were obtained by considering multiple regions (see methods) and are not specific to the brain region selected for WGS. Mean and standard deviation are shown for continuous variables. Absolute frequency and percentage are shown for categorical variables

### Mayo and MSBB cohorts

Samples included in the Mayo case-control study were obtained either from the Mayo Brain Bank or from the Banner Sun Health Institute and classified as control, AD, progressive supranuclear palsy (PSP) or pathologic aging based on neuropathological assessment [31]. All AD samples demonstrated tau pathology (Braak score ≥ 4). All controls demonstrated no or minimal tau pathology (Braak score ≤ 3) and were without any other neurodegenerative disease. In this study, samples from the Banner Sun Health Institute were excluded since all AD samples were obtained from the Mayo Brain Bank and we observed a difference in mtDNAcn between control samples from the two different centers. Since age at death was right-censored at 95 years for HIPPA compliance, we stratified age into 5 year strata with an open interval > 95 years for our analyses. Specimens for WGS were sampled from the temporal cortex (TCX) (*n* = 262). Sample characteristics are summarized in Table S1.

The Mount Sinai Brain Bank (MSBB) case-control study cohort was assembled after applying stringent inclusion/exclusion criteria and represents the full spectrum of cognitive and neuropathological AD severity in the absence of discernable non-AD neuropathology [32]. Neuropathological assessments were performed according to the Consortium to Establish a Registry for Alzheimer’s Disease (CERAD) protocol and included assessment by hematoxylin and eosin, modified Bielschowski, modified thioflavin S, and anti-amyloid-β (4G8), anti-tau (AD2) and anti-ubiquitin (Dakoa Corp.). Pathologic AD was defined based on the CERAD stages definitive AD and probable AD. Samples staged as possible AD or not AD were considered as controls. Age at death was right-censored and stratified as for the Mayo cohort. Specimens for WGS were sampled from the frontal pole (FP) (*n* = 337). Sample characteristics are summarized in Table S2. A total of *n* = 67 samples were obtained from non-Caucasians and excluded from the mtDNAcn GWAS and mtDNA heteroplasmy analyses.

### Whole-genome sequencing

WGS libraries from all three studies were prepared using the KAPA Hyper Library Preparation Kit in accordance with the manufacturer’s instructions. Briefly, 650 ng of DNA was sheared using a Covaris LE220 sonicator (adaptive focused acoustics). DNA fragments underwent bead-based size selection and were subsequently end-repaired, adenylated, and ligated to Illumina sequencing adapters. Final libraries were evaluated using fluorescent-based assays including qPCR with the Universal KAPA Library Quantification Kit and Fragment Analyzer (Advanced Analytics) or BioAnalyzer (Agilent 2100). Libraries were sequenced on an Illumina HiSeq X sequencer (v2.5 chemistry) using 2 × 150 bp cycles.

### Variant calling

Sequence alignment and nuclear DNA variant calling was performed by the automated pipeline of the New York Genome Center, where all samples were sequenced [33]. Briefly, paired-end 150 bp reads were aligned to the GRCh37 human reference using the Burrows-Wheeler Aligner (BWA-MEM v0.7.8) and processed using the GATK best-practices workflow that includes marking of duplicate reads using Picard tools v1.83, local realignment around indels, and base quality score recalibration (BQSR) using the Genome Analysis Toolkit (GATK v3.4.0).

Mitochondrial homo- and heteroplasmic single-nucleotide variants and small indels (< 50 bp) were called following GATK’s Best Practices Mitochondria Pipeline 1.1.0 (https://github.com/gatk-workflows/gatk4-mitochondria-pipeline). GATK v4.1.2 was used to run the pipeline with the rCRS (NC_012920.1) as mtDNA reference sequence. Briefly, sequence reads (mapped to MT or unmapped in original bam file) were mapped to the rCRS and to the rCRS shifted by 8000 base pairs using BWA-MEM v0.7.8. Variants were detected using GATK’s Mutect2 in both bam files. Subsequently, variants were merged into one VCF file using the shifted rCRS for variants around the artificial start/end position of the circular genome and the unmodified rCRS for the remaining part the MT genome. Variant filtering included the median autosomal chromosome coverage to filter potential polymorphic nuclear mitochondrial DNA (NUMT) variants, the mtDNA contamination estimated by the haplochecker (mitolib 0.1.2) to account for possible contamination, a minimum minor allele frequency of 0.03, and an F-score beta of 1 (default settings).

### Estimation of the mtDNAcn from WGS data

**Fig. 1:**
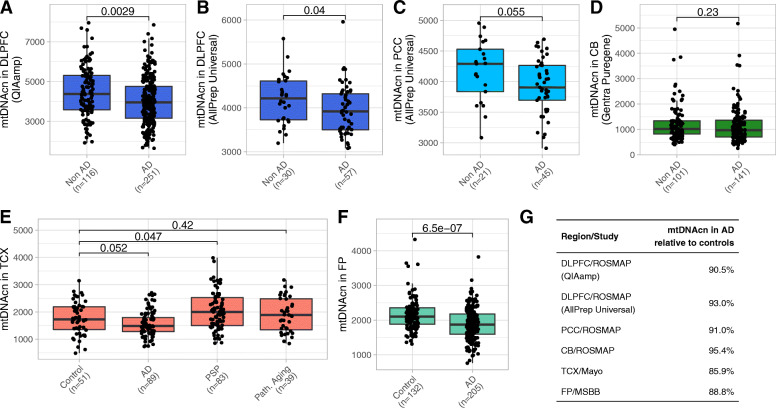
The mtDNAcn is reduced in cortical brain regions in AD. **(A-D)** Boxplots comparing the mtDNAcn in the DLPFC (A and B), in the PCC (C), and in the CB (D) from individuals of the ROSMAP cohort with and without pathologic AD diagnosis. The used DNA extraction kit is denoted in brackets on the y axis. Wilcoxon rank-sum test was applied to calculate p values. **(E)** Boxplot shows the mtDNAcn in the TCX of controls, AD cases, PSP cases, and cases of pathologic aging from the Mayo study. Wilcoxon rank-sum test was applied to calculate *p* values. **(F)** Boxplot shows the mtDNAcn in the FP of controls and AD cases from the MSBB study. Wilcoxon rank-sum test was applied to calculate p values. **(G)** The estimated relative mtDNAcn observed in AD compared to controls is shown for each brain region and study

The median sequence coverages of the autosomal chromosomes *cov*_*nuc*_ and of the mitochondrial genome *cov*_*mt*_ were calculated using R/Bioconductor (packages GenomicAlignments and GenomicRanges). Ambiguous regions were excluded using the intra-contig ambiguity mask from the BSgenome package. The mtDNAcn was defined as (*cov*_*mt*_/*cov*_*nuc*_) × 2. Raw mtDNAcn was used for the first analysis shown in Fig. 1. For subsequent analyses the mtDNAcn was z-standardized within each brain region and DNA extraction kit and then logarithmized. The normalization facilitated the combined analysis of the two different kits used for the DLPFC and resulted in approximately normal mtDNAcn measures (Fig. S1).

### Estimation of the neuronal proportion from RNA-seq data

Proportion of neurons were estimated for *n* = 327 DLPFC samples with RNA-seq data by applying the Digital Sorting Algorithm (DSA) [34] to a set of published marker genes that were previously used to deconvolute cortical RNA-seq data [35]. RNA-seq data were TMM normalized and technical variables were regressed out. Only marker genes with a mean transcription level ≥ 2 cpm in our dataset were used (87 markers for astrocytes, 88 for endothelial cells, 59 for microglia, 90 for neurons, and 86 for oligodendrocytes). As proposed by Wang, Allen [35], DSA was modified so that the median instead of the mean transcription level of all marker genes per cell type was calculated.

### Estimation of mitochondrial content from proteomic data

Tandem mass tag (TMT) multiplexed mass spectrometry data was available for a subset of *n* = 156 DLPFC samples that also had WGS data. Mass spectrometry data was preprocessed and normalized as previously described [36]. Ten proteins were selected to quantify mitochondrial mass. Six proteins (CS, LRPPRC, SLC25A24, TIMM44, GCDH, and TRAP1) were taken from the Human Protein Atlas’ list of mitochondrial marker proteins [37]. The four additional proteins (HSPD1, VDAC2, VDAC3, and TOMM20) have been previously used as mitochondrial markers and confirmed to be specific to mitochondria in the Human Protein Atlas. Fig. S5A shows the correlation between the 10 selected proteins. The median protein level of these ten proteins was used as mitochondrial content measure. Similarly, the respiratory chain complexes I-V were quantified by calculating the median level of all proteins that were detected in our TMT data and were known subunits or assembly factors of the respective complex. Supplementary Excel File 3 lists the proteins used to calculate the scores. This list includes 11 of the 13 mtDNA-encoded proteins (see column C in Supplementary Excel File 3) and their median protein level was used to specifically quantify the abundance of mtDNA-encoded proteins.

### Statistical methods

Statistical analyses were conducted in R. Standard multivariable linear regression models were used when the standardized mtDNAcn or cognitive function was the dependent variable. Quasi-Poisson regression models were used when the number of mtDNA heteroplasmic mutations (heteroplasmy level) was the dependent variable. Reported *p* values were obtained from likelihood ratio tests and unadjusted unless stated otherwise in the text. Continuous variables were standardized to obtain comparable effect sizes unless stated otherwise. All variables included in the respective models are described in the text and figures. When analyzing the effect of SNPs on mtDNAcn, population structure was modeled by the first three principle components obtained from the genotypes after pruning. Random effects meta-analyses were performed using the R package meta v4.13. *P* values and quasi-Bayesian confidence intervals for the mediation analysis were calculated using the R package mediation v4.5.0 [38]. The sparse Gaussian graphical model (Fig. 5B) was estimated by the graphical LASSO method with EBIC model selection and a default tuning parameter of 0.5 as implemented in the R package bootnet [39]. The nonparanormal transformation implemented in the package huge was applied to relax the assumption of normality for the mtDNA heteroplasmy levels before estimating the network. Network stability was assessed using 1000 bootstraps.

## Results

### mtDNAcn is reduced in the AD cortex

Whole genome sequencing (WGS) data were generated from *n* = 762 post-mortem brains from ROSMAP, two harmonized cohort studies of aging and dementia [19]. Brain specimens were obtained from the dorsolateral prefrontal cortex (DLPFC) (*n* = 454), the posterior cingulate cortex (PCC) (*n* = 66), and the cerebellum (CB) (*n* = 242) from different individuals. Table 1 summarizes pathologic and demographic information of the samples. The number of aligned sequence reads per sample ranged from 643.5 × 10^6^ to 1475.0 × 10^6^ reads (median of 899.1 × 10^6^ reads) (Supplementary Excel File 1). The mtDNAcn was estimated from the WGS data by dividing the median coverage of the MT chromosome by the median coverage of the autosomal chromosomes and multiplying the ratio by two [40, 41].

We first studied whether the mtDNAcn in the three selected brain regions was altered in individuals with pathologic AD diagnosis compared to those without AD (Fig. 1A-D). Two different DNA extraction kits were used for the DLPFC samples. Since the DNA extraction method can influence the mtDNAcn estimates [42, 43], the DLPFC samples were split by kit and analyzed separately. In line with previous reports [8, 9], we found a lower mtDNAcn in AD in the DLPFC (*p* = 0.0029 and *p* = 0.040 for the two kits/batches, Wilcoxon rank-sum tests) and also in the PCC (*p* = 0.055, Wilcoxon rank-sum test). Changes in the PCC were of similar magnitude but failed to reach significance probably due to the smaller sample size. Changes in the CB were not significant.

Next, we analyzed the mtDNAcn in two additional brain regions using public WGS data from the Mayo Clinic study and the Mount Sinai Brain Bank (MSBB) study (Tables S1,S2). Both regions, the temporal cortex (TCX) in the Mayo data (*p* = 0.052, Wilcoxon test) and the frontal pole (FP) in the MSBB data (*p* = 6.5 × 10^− 7^, Wilcoxon test), demonstrated a lower mtDNAcn in AD samples compared to control samples (Fig. 1E,F). The reduction of mtDNAcn in AD ranged from 7.0 to 14.2% across the cortical regions from the three studies (Fig. 1G). The Mayo study includes samples diagnosed with pathologic aging, which is characterized by amyloid-β loads at similar levels as in AD and an absence of tau pathology. Whether pathologic aging is an early stage of AD or whether these individuals have protective factors that prevent the development of tau pathology is unknown [44]. We did not observe altered mtDNAcn levels in the TCX of individuals with pathologic aging (Fig. 1E). Additionally, the Mayo study includes samples with progressive supranuclear palsy (PSP). PSP is a tauopathy primarily characterized by tau inclusions in the brain stem and subcortical neurons. Interestingly, we observed a moderately higher mtDNAcn in the TCX of the PSP samples compared to the control samples (*p* = 0.047, Wilcoxon rank-sum test) (Fig. 1E).

For the subsequent analyses, we log-transformed the raw mtDNAcn and calculated z-scores for each of the six datasets shown in Fig. 1A-F. Then, we merged the two ROSMAP DLPFC datasets generated by different DNA extraction kits and verified that the normalized mtDNAcn approximately follows a standard normal distribution (Fig. S1). We note that the raw mtDNAcn before normalization should also be considered as a relative rather than an absolute measurement since the estimations are likely affected by experimental factors, which impedes a comparison across different brain regions or studies when different protocols and reagents were used.

### Lower mtDNAcn is primarily related to tau in the DLPFC and to TDP-43 in the PCC

**Fig. 2:**
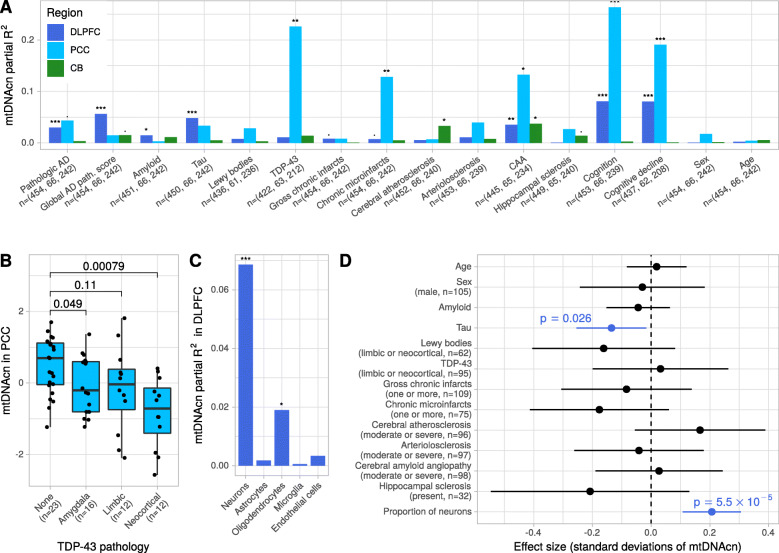
Changes of the mtDNAcn are primarily associated with tau in the DLPFC and TDP-43 in the PCC. **(A)** The bars indicate the mtDNAcn’s variance explained (partial R^2^) by different pathologies, cognitive measures, and demographics in the ROSMAP cohort. Each Variable was analyzed separately in a regression model with mtDNAcn as outcome adjusted for sex and age. The cognitive variables were additionally adjusted for education. The results for sex and age were obtained from a model adjusted for global AD pathology. Colors indicate brain regions. Asterisks indicate significance levels obtained by F-tests (*** for *p* ≤ 0.001, ** for *p* ≤ 0.01, * for *p* ≤ 0.05, and · for *p* ≤ 0.1). The sample size available for each pathology and brain region is denoted on the y axis. **(B)** Boxplot shows the mtDNAcn in the PCC for different stages of TDP-43 pathology. Wilcoxon rank-sum test was used to calculate p values. **(C)** The bars indicate the mtDNAcn’s variance explained (partial R^2^) by cell type proportions estimated from RNA-seq data in a subset of *n* = 327 DLPFC samples. Each cell type proportion was analyzed separately in a regression model with mtDNAcn as outcome adjusted for sex and age. Significance levels were obtained and indicated by asterisks as in (A). **(D)** Forest plot shows the result from a multivariable regression model with mtDNAcn in the DLPFC as outcome and the pathologic and demographic variables denoted on the y axis as explanatory variables. Estimated coefficients are shown as dots and the line segments represent the respective 95% confidence intervals. Continuous variables were z-standardized. Categorical variables were dichotomized and the factor level and case numbers corresponding to the plotted coefficient are denoted in brackets under the variable name. t-test was applied to calculate p values. A total of *n* = 288 cases with complete observations of all variables were used to fit the model

AD pathology is the most common brain pathology in the aged brain but is often accompanied by comorbid brain pathologies. To determine which pathological feature is driving the association with mtDNAcn, we first analyzed each of the 12 pathologic variables shown in Fig. 2A as well as cognition and cognitive decline separately adjusted only for age and sex. The two cognitive variables were additionally adjusted for education. Further, we also analyzed the association with age and sex adjusted for AD pathology (Fig. 2A). Detailed results of these analyses are given in Table S3 and can be summarized by four main findings: First, in the DLPFC, mtDNAcn is mainly associated with tau (*p* = 2.9 × 10^− 6^, t test) rather than amyloid-β pathology (*p* = 0.011, t test) and is also associated with cognition proximate to death (*p* = 7.9 × 10^− 10^, t test) and cognitive decline (*p* = 1.8 × 10^− 9^, t test). Second, in the PCC, we also found an association with cognition (*p* = 1.7 × 10^− 5^, t test) and cognitive decline (*p* = 5.5 × 10^− 4^, t test), but the association with tau was not significant probably due to the smaller sample size. Interestingly, we found an association with TDP-43 pathology (*p* = 2.1 × 10^− 3^, F test) in the PCC, which explained 23% of the variance in mtDNAcn. Third, in the CB, neither pathologies nor cognitive measures were associated with mtDNAcn. Fourth, neither age nor sex were associated with mtDNAcn in any of the three regions when adjusting for AD pathology. All significant pathologies demonstrated an inverse correlation with mtDNAcn, and a lower mtDNAcn was associated with lower cognitive performance and a steeper rate of cognitive decline, consistent with the notion that a high mtDNAcn is a feature of healthy mitochondrial function in the aged brain.

Pathologic TDP-43 is known to localize inside mitochondria and has recently been described to trigger the release of mtDNA molecules from the mitochondrial matrix into the cytoplasm in cellular systems [45–47]. In our study, limbic-predominant age-related TDP-43 encephalopathy neuropathological change (LATE-NC) was captured by phosphorylated TDP-43 immunohistochemistry, and staged according to the recent consensus working group report (stage 0, no TDP-43; stage 1, localized to amygdala; stage 2, extension to hippocampus or entorhinal cortex; stage 3, extension to the neocortex) [48]. In the PCC, we observed a gradual reduction of mtDNAcn across the stages with a distinctly lower mtDNAcn in stage 3 (Fig. 2B). In contrast, in the DLPFC we observed only a minor reduction in the latest stage (Fig. S2A). Our findings are consistent with previous reports of prominent PCC hypometabolism in LATE-NC [49], and DLPFC being involved only in the most advanced stage of LATE-NC [50, 51]. Thus, mtDNAcn alterations in the PCC might be an early feature of LATE-NC, even though the current LATE-NC staging scheme does not consider the PCC.

Neurons have relatively high concentrations of mitochondria, and changes in the proportion of neurons during the course of AD can confound mtDNAcn measurements at the tissue level. We therefore estimated the proportions of neurons and of glial cell types from DLPFC RNA-seq data for a subset of *n* = 327 samples with DLPFC mtDNAcn estimates. As expected, the neuronal proportion was significantly positively associated with mtDNAcn, accounting for 6.9% of the variance in mtDNAcn (*p* = 1.7 × 10^− 6^, t test) (Fig. 2C, Table S4). We also found an association with oligodendrocytes (*p* = 0.013, t test), but in contrast to the neuronal proportion this association disappeared when we modeled the different cell types together, indicating that adjusting for neuronal proportions in downstream analyses is sufficient.

Since many of the pathologies are correlated with each other, we next modeled all pathologies, age, sex and neuronal proportion in a multivariable regression model to identify the primary driver of mtDNAcn changes in the DLPFC. As shown in Fig. 2D, only tau pathology and neuronal proportion remained significantly associated with mtDNAcn when accounting for all pathologies. Thus, our data suggest that tau burden is directly associated with lower mtDNAcn in the DLPFC and that this relationship cannot be explained by neuronal loss alone. Similar results for tau were obtained from a model unadjusted for neuronal proportion (Fig. S2B).

### mtDNAcn is associated with cognitive function independent of brain pathologies

**Fig. 3:**
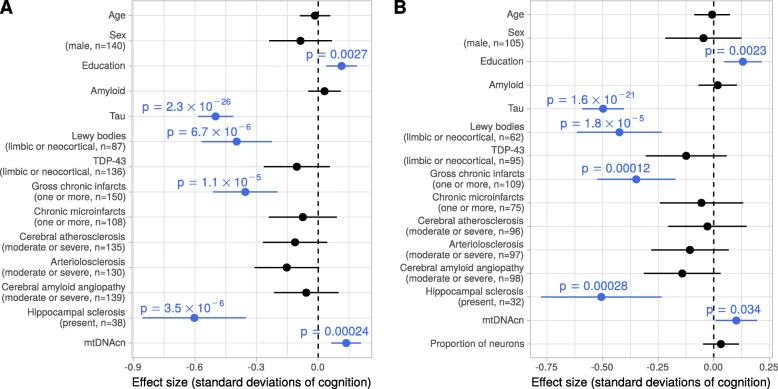
The mtDNAcn is associated with cognitive function independent of brain pathologies. **(A)** Forest plot shows the result from a multivariable regression model with global cognitive function as outcome and the pathologic variables, demographic variables and mtDNAcn as explanatory variables (y axis). Estimated coefficients are shown as dots and the line segments represent the respective 95% confidence intervals. Continuous variables were z-standardized. Categorical variables were dichotomized and the factor level and case numbers are denoted in brackets under the variable name. t-tests were applied to calculate p values. A total of *n* = 393 cases with complete observations of all variables were used to fit the model. **(B)** Same plot as in (A) but with neuronal proportion as additional variable in the model reducing the number of samples with complete observations to *n* = 287

The univariate analysis (Fig. 2A) revealed that a lower mtDNAcn in the cortex is associated with a decline of cognitive function. We next sought to investigate whether this relation can be explained by the correlation of mtDNAcn with tau, or whether mtDNAcn and tau have effects on cognitive functioning independent of each other. We modeled cognitive function as outcome depending on age, sex, education, mtDNAcn, and ten neuropathologies including tau using our DLPFC data. In this model, mtDNAcn was significantly associated with cognitive function (*p* = 2.4 × 10^− 4^, t test) together with tau, Lewy bodies, gross chronic infarcts, hippocampal sclerosis, and education (Fig. 3A). Tau was the most important predictor for cognitive function. When we added neuronal proportion to the model, the effect size of mtDNAcn was attenuated by 26% but remained significant (*p* = 0.034, t test) despite the smaller number of samples that have both measurements neuronal proportion and mtDNAcn (Fig. 3B). These results indicate that the observed changes in the mtDNAcn in the DLPFC capture aspects of mitochondrial health beyond neuronal loss and correlates with cognitive function independent of pathologies.

### Genetic determinants of the mtDNAcn

Two recent genome-wide association studies (GWAS) of blood samples from the UK Biobank identified 96 and 50 independent loci respectively that affected the mtDNAcn in whole blood [52, 53]. Functional annotation of some loci pointed to blood-specific pathways such as platelet activation or megakaryocyte proliferation, an association consistent with the influence of platelets on whole blood mtDNAcn [54], whereas other loci were located at genes involved in mitochondrial pathways and therefore could regulate the mtDNAcn in brain tissues as well. Since our sample size is limited for a GWAS, we conducted a focused analysis of 81 lead SNPs identified in the prior study [52] and with a minor allele frequency ≥ 5% in our data. We separately analyzed the four brain regions with at least 100 samples (DLPFC, CB, TCX, and FP) and subsequently performed a random effects meta-analysis. A total of *n* = 67 non-Caucasian individuals in the MSBB dataset (FP) were excluded resulting in *n* = 1228 brain samples. The association tests were adjusted for age, sex, population structure, and quantitative AD pathology score (DLPFC, CB) or post-mortem diagnosis respectively (TCX, FP). After Bonferroni adjustment, only the top SNP rs11085147 from the original study was significantly associated with mtDNAcn in our brain data (p_BF_ = 0.0436). The direction of the effect was identical in all four brain regions and consistent with the originally reported direction in blood. Each additional dosage of the alternative allele increased the mtDNAcn by 0.23 standard deviations. The SNP rs11085147 is a missense variant of the gene Lon Peptidase 1, Mitochondrial (*LONP1*), which binds mitochondrial DNA and is involved in mtDNA replication and mitogenesis [55]. rs11085147 has not been linked to AD risk in genome-wide association studies of AD. Detailed results for all 81 SNPs are given in Supplementary Excel File 2.

The strongest genetic risk factor for late-onset AD is the Apolipoprotein E (*APOE*) locus. Several studies using mouse models or human brain implicated the ε4 risk allele with mitochondrial dysfunction [56, 57]. We therefore analyzed the effect of the *APOE* ε4 allele on the mtDNAcn in our four datasets with ≥100 samples. The *APOE* ε4 allele was associated with lower mtDNAcn (*p* = 8.0 × 10^− 7^, random effects meta-analysis), but this association was attenuated considerably when adjusting for pathologies (*p* = 0.014, random effects meta-analysis) (Table 2) suggesting that a large fraction but not the complete *APOE* ε4 effect on mtDNAcn is mediated via AD pathology. Indeed, mediation analysis of the DLPFC samples (Fig. S3) revealed that 44% of the total effect is mediated via pathology.

**Table 2:** Effect of *APOE* ε4 genotype on mtDNAcn

Region/Study	N of *APOE* ε4 dosages (0, 1, 2)	Unadjusted for pathology			Adjusted for pathology*		
		β	SE	p	β	SE	p
DLPFC/ROSMAP	335, 112, 7	−0.364	0.099	2.8 × 10^− 04^	−0.204	0.105	0.051
CB/ROSMAP	182, 56, 4	−0.292	0.134	0.031	−0.223	0.144	0.124
TCX/Mayo	183, 70, 9	−0.280	0.114	0.015	−0.126	0.121	0.301
FP/MSBB	171, 88, 11	−0.158	0.106	0.137	−0.048	0.104	0.642
Meta-analysis	871, 326, 31	−0.275	0.056	8.0 × 10^− 07^	−0.141	0.058	0.014

*ROSMAP was adjusted by adding a quantitative score for amyloid and tau burden to the model. Mayo and MSBB were adjusted by adding the pathologic diagnosis to the model

### mtDNA heteroplasmy levels in the cortex are higher with age

**Fig. 4:**
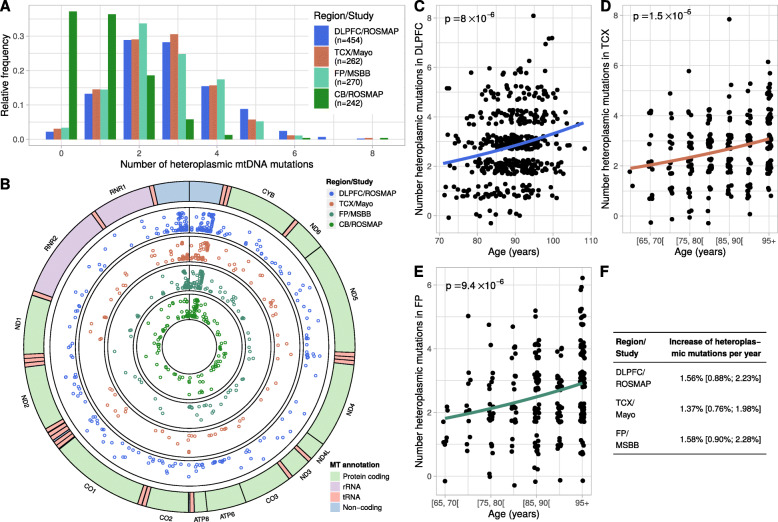
Frequency of mtDNA heteroplasmic mutations in cortical regions increases with age. **(A)** Histogram depicts the number of mtDNA heteroplasmic mutations detected per sample in each of the four studied brain regions. **(B)** Circular plot of the mitochondrial genome shows the genome annotation on the outer circle. The four inner circles show the genomic locations and the relative frequencies (y-axis) of mtDNA heterplasmic mutations detected in each of the four brain regions. **(C-E)** Scatter plots showing the relation between age and mtDNA heteroplasmy burden in the DLPFC (C), in the TCX (D), and in the FP (E). Some jitter was added before plotting the points to avoid overlapping points. Regression curves and p values were obtained from quasi-Poisson regression models with the number of mtDNA heteroplasmic mutations as outcome and age as independent variable. In the TCX and FP, age was grouped into 5-year strata because of censored data. **(F)** Table shows the effect sizes of age on mtDNA heteroplasmy burden obtained from the Quasi-Poisson regression models depicted in panels (C-E). The 95% confidence intervals are shown in brackets

We detected the number of mtDNA heteroplasmic point mutations and small indels (less than 50 bp) in the WGS data to assess the relation of mtDNA mutational burden (heteroplasmy level) with mtDNAcn and pathologies. Here, we defined a mtDNA heteroplasmic mutation as a point mutation or small indel with a relative frequency between 3 and 90%. As in the previous section, we studied the four brain regions with ≥100 samples and excluded the non-Caucasian samples from the MSBB dataset (a total of *n* = 1228 brain samples). On average, between 2.6 and 2.8 heteroplasmic mutations were observed in the three cortical regions, whereas the CB demonstrated less heteroplasmic mutations (mean of 1.0) (Fig. 4A). As expected, most of the heteroplasmic mutations were located in the mtDNA hypervariable control region, also known as the D-loop (Fig. 4B). None of the genes encoded in the mitochondrial genome showed an enrichment of heteroplasmic mutations.

Next, we used the DLPFC dataset to study whether the mtDNA heteroplasmy levels are related to brain pathologies, cognitive function, sex, age, and mtDNAcn. In contrast to the mtDNAcn, heteroplasmy levels were not associated with AD pathologies or cognitive function. We also found no significant association between heteroplasmy levels and mtDNAcn in the DLPFC. However, heteroplasmy levels increased significantly with advancing age: we report estimated 1.6% increase per year in the DLPFC (*p* = 8.0 × 10^− 6^, quasi-Poisson regression) (Fig. 4C). The accumulation of mtDNA heteroplasmic mutations with aging in the cortex were replicated in the TCX and FP data with similar effect sizes (Fig. 4D-F). In line with the low abundance of heteroplasmic mutations in the CB, the association with age was not detectable in this brain region. The association with age persisted when we adjusted for pathologic diagnosis, sex, and mtDNAcn using multivariable models for each of the three regions DLPFC, TCX, and FP (Fig. S4, Table S5). In addition, we found a weak association with AD diagnosis and with mtDNAcn in the TCX, which were absent in the DLPFC and in the FP. Overall, our analyses showed that age is the primary driver of mtDNA heteroplasmic point mutations and small indels in the cortical regions and that the CB demonstrates low mtDNA mutation rates.

### Altered mtDNAcn does not necessarily imply alteration in mitochondrial content

**Fig. 5:**
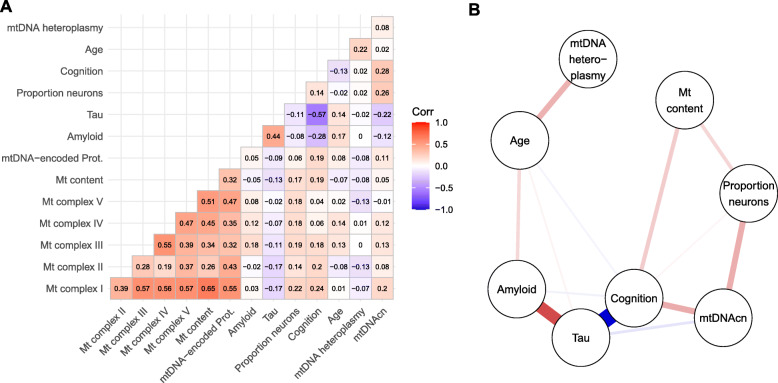
Correlates of mitochondrial health demonstrate complex relationship with AD-related traits in the DLPFC. **(A)** Heatmap shows pairwise Pearson correlations between scores for the abundance of mitochondrial complexes I to V, mitochondrial content, and mtDNA-encoded proteins derived from mass spectrometry data, amyloid and tau derived from immunohistochemistry data, proportion of neurons derived from RNA-seq data, cognitive function, age, mtDNA heteroplasmy levels and mtDNAcn. Nonparanormal transformation was applied to mtDNA heteroplasmic mutation counts before calculating correlations. Number of available cases for each pair of variables is given in Fig. S4B. **(B)** Graph shows a sparse representation of the partial correlations between the different variables. An edge in this graph indicates that the two connected variables are correlated with each other after controlling for all other variables in the graph. Thickness and color of the edges represent the strength and direction of the partial correlation

A lower mtDNAcn in a cell can reflect a lower mtDNAcn per mitochondrion or a lower mitochondrial content in the cell. We therefore quantified the mitochondrial content in the DLPFC using mass spectrometry-based proteomics data for a subset of 156 subjects who have DLPFC mtDNAcn measurements. Abundances of 10 proteins specific for mitochondria selected from the Human Protein Atlas were quantified, and the median of the standardized protein values was used as a mitochondrial content score (Fig. S5A) [37]. Interestingly, we found no significant correlation between mtDNAcn and mitochondrial content, but both measures were positively correlated with neuronal proportion and cognitive function (Fig. 5A, Fig. S5B). This suggests that these two measures (mtDNAcn and protein-based content) represent different aspects of mitochondrial health that both affect cognition but can change independently of each other. A similar lack of correlation between mtDNAcn and mitochondrial content has been previously reported for muscle tissue [58, 59]. Next, we derived a similar score using respiratory chain proteins encoded by the mitochondrial genome (data available for 11 of the 13 proteins, see methods, Supplementary Excel File 3) and observed a weak positive non-significant correlation of 0.11 with mtDNAcn (Fig. 5A), which is consistent with the notion that a moderate loss of mtDNA in not sufficient to reduce mitochondrial protein expression significantly. We also used our mass spectrometry data to quantify the abundances of respiratory chain complexes (see methods, Supplementary Excel File 3). Protein levels for complexes I to V were highly correlated with each other and with mitochondrial content, but demonstrated only a weak correlation with mtDNAcn (Fig. 5A). To further disentangle the relationship between mtDNAcn, mitochondrial content, mitochondrial heteroplasmy levels and AD-related phenotypes, we estimated the partial correlations between these variables. The graph in Fig. 5B represents a sparse representation of the partial correlation matrix of the variables. An edge in this graph represents a direct association between two variables that remained when controlling for all other variables in the graph. The stability of the graph’s edges was assessed using bootstrapping (Fig. S5C). The top left of the graph shows age as the primary driver for amyloid-β and mtDNA heteroplasmy levels. The latter are not associated with any other variable in our graph suggesting that heteroplasmic mtDNA point mutations and small indels are not involved in AD pathogenesis. In the lower part of the graph, amyloid-β is strongly associated with tau, and tau with cognitive function. Tau is also associated with mtDNAcn as suggested by our previous analyses. mtDNAcn and mitochondrial content are not directly connected, but both are associated with neuronal proportion and cognitive function. Together, these findings indicate a complex involvement of mitochondria in neurodegeneration and the need for future studies to measure both parameters to better understand mitochondrial recalibrations in AD.

## Discussion

We characterized the mtDNAcn in 1361 aged human brain samples from five regions and estimated a reduction of mtDNAcn by 7 to 14% in pathologic AD compared to non-AD samples in the cortical regions profiled in this study. We then leveraged the detailed pathologic and cognitive characterization of the ROSMAP study to identify the primary drivers of mtDNAcn loss and to assess the relation to cognitive function in the presence of mixed pathologies, which are frequently observed in the aged human brain [20].

In the DLPFC, lower mtDNAcn was primarily associated with tau pathology. When accounting for ten common brain pathologies, tau was the only pathology that remained significantly associated with mtDNAcn. The mtDNAcn at the tissue level depends on the cell type composition of the studied tissue, as is well known in blood [54]. We therefore estimated the neuronal proportion in the DLPFC samples, confirmed that the neuronal proportion is associated with mtDNAcn, and demonstrated that the association between tau pathology and mtDNAcn was still significant - albeit attenuated - when adjusting for neuronal proportion. Although previous studies showed that informative estimates of neuronal proportions can be obtained from DLPFC RNA-seq data [60], we cannot exclude that changes in the complex composition of heterogeneous brain cells still contribute to the observed association. For example, a tau-mediated preferred depletion of a specific neuronal cell subtype could alter the composition within the neuronal cell population and cause changes in the mtDNAcn at the tissue level. Future single-cell studies will have to show to what extent the tau-related mtDNAcn loss observed in this study is a cell-intrinsic feature and which cell subtypes are affected.

Nevertheless, our analysis adjusted for neuronal proportions suggests that the relationship is not merely caused by tau-driven loss of mitochondria-rich neurons; other mechanisms link tau pathology to reduced mtDNAcn. Mechanisms that could potentially underlie our observation have been explored in model systems. For example, hyperphosphorylated tau has been shown to impair mitochondrial axonal transport [61, 62] and to affect mitochondrial fission/fusion dynamics [63, 64]. Conversely, reduced mtDNAcn has been shown to promote tau oligomerization in human neuronal cell lines [65], suggesting complex interactions between tau and mitochondria. Tau is also known to be a strong predictor for cognitive decline. Interestingly, when studying cognitive function, mtDNAcn was a significant predictor in our model that included tau, nine other brain pathologies, neuronal proportion, and demographic variables.

In contrast to tau pathology, amyloid-β pathology was not significantly associated with mtDNAcn after accounting for other pathologies. This finding was supported by the results from the Mayo study where the mtDNAcn was not reduced in persons diagnosed with pathologic aging, which is defined by high amyloid-β burden but no or minimal tau pathology [44]. Interestingly, numerous studies have demonstrated that the amyloid-β precursor protein (APP) as well as amyloid-β peptides localize at mitochondria and affect mitochondrial function and bioenergetics [66, 67]. Further, C-terminal fragments resulting from the processing of APP (APP-CTFs) have been implicated in AD and may trigger morphological and functional changes of mitochondria [68]. The absence of an association in our data could result from measuring only amyloid-β peptides (1–40) and (1–42) (both were detected by our antibody), which does not fully capture APP processing. Further, amyloid-β could still impair mitochondrial respiratory chain capacity or other non-energetic functions (e.g. calcium handling) without changes in mtDNAcn.

In the PCC, TDP-43 pathology was the most important factor and explained 23% of the mtDNAcn’s variance. The effect of TDP-43 on mitochondria has been mainly studied in model systems for ALS, where suppressing the localization of TDP-43 inside mitochondria reduces TDP-43 toxicity [46]. When accumulating inside mitochondria, TDP-43 induces the release of mtDNA into the cytoplasm via the permeability transition pore [47]. Whether any of these mechanisms underlie the correlation observed in our post-mortem brain data remains to be elucidated. Interestingly, the effect of TDP-43 pathology on mtDNAcn was moderate in the DLPFC, which could be caused by the distinct spatial pattern of TDP-43 progression in the aged brain or by a larger susceptibility of the neurons in the PCC to TDP-43 pathology.

The mtDNAcn has been suggested as a biomarker for aging because several studies of peripheral blood have reported an inverse correlation between age and mtDNAcn [69, 70]. However, more recent studies demonstrated that the inverse correlation is likely caused by unaccounted age-related changes of cell type proportions in the blood [71, 72]. In our study, we found no association between mtDNAcn and age or sex in the studied brain regions when we adjusted for pathologies. We also found no significant associations in the CB, which accumulates much less AD pathology than the DLPFC or the PCC. In summary, our results indicate that lower mtDNAcn is driven by certain pathologies rather than aging and restricted to brain regions directly affected by the respective pathologies. Similarly, a study of Parkinson’s disease brains found lower mtDNAcn in the vulnerable substantia nigra but not in the frontal cortex, which is less affected in Parkinson’s disease [73].

To assess the effect of genetic variants on the mtDNAcn in the brain, we performed a targeted analysis of 81 lead SNPs that were recently reported by a large GWAS of mtDNAcn in blood [52]. A missense variant in the protease *LONP1* demonstrated a moderate but significant effect size of 0.23 standard deviations larger mtDNAcn per dosage of the alternative allele in our meta-analysis of four brain regions. The original blood-based GWAS also detected a single variant at the *APOE* locus, which harbors the strongest genetic risk factor for AD. We therefore investigated the effect of the *APOE* ε4 haplotype (defined by two coding variants) on the mtDNAcn. While a large fraction of the *APOE* ε4 effect was mediated via AD pathologies, we also found evidence for a direct effect on the mtDNAcn. These findings support the hypothesis that the *APOE* ε4 allele exerts its risk not only via regulating amyloid-β aggregation and clearance but also through other pathways, including mitochondrial bioenergetics [74, 75]. In summary, while this study is the first, to our knowledge, to report evidence for genetic regulation of the mtDNAcn in the brain, the sample size was a limiting factor and future non-targeted studies with much larger sample sizes will likely detect more loci.

Several tissues in addition to brain accumulate mtDNA mutations during aging [76–78]. Here, we analyzed point mutations and small indels and found higher heteroplasmy levels with age in three cortical regions and estimated an association consistent with an accumulation rate of about 1.5% per year in this age group. We did not find a significant association with age in the CB where the overall frequency of mtDNA heteroplasmic mutations was very low (mean of 1.0) supporting the theory of region-specific accumulation of mtDNA mutations in the brain [79]. Consistently, a previous study [9] failed to detect an association between age and heteroplasmy levels in their samples which were mainly (87%) obtained from the CB. Thus, the CB seems to acquire less heteroplasmic mutations than the cortex or the heteroplasmic mutations in the CB have, on average, a lower relative frequency which may often not surpass the detection threshold of 3% used in this study. In contrast to the mtDNAcn, we found little evidence for the involvement of heteroplasmic mtDNA point mutations and small indels in AD or in the development of other brain pathologies. However, many of the previous studies reporting associations between mtDNA heteroplasmy and neurodegenerative diseases investigated structural variants, primarily large-scale mtDNA deletions, which were not considered in this study but are more likely to be pathogenic than small mutations [17, 18]. Overall, our work showed that small mtDNA heteroplasmic mutations accumulate over an individual’s life-time in the cortex but are not related to neurodegenerative diseases. The seeming insignificance of low-level mtDNA heteroplasmy is consistent with a large reserve respiratory capacity, such that mtDNAcn is generally in excess (> 50%) of the minimum number of mtDNA copies required to sustain bioenergetic capacity [80].

A lower mtDNAcn can be caused by lower mitochondrial mass in the cells or by a lower mtDNAcn per mitochondrion. We generated a proteomic measure of mitochondrial content for a subset of our DLPFC samples and showed that the mtDNAcn and mitochondrial content vary relatively independent of each other in the DLPFC. The decoupling of the two variables may be explained by distinct mechanisms that regulate mtDNAcn and mitochondrial content and that have been successfully manipulated in model systems to regulate one of the readouts, without affecting the other one [5]. Similarly, the correlation between the abundance of mtDNA-encoded proteins and mtDNAcn was weak, and protein measures of the respiratory chain complexes were highly correlated with each other and with mitochondrial content protein markers, but showed only weak correlations with mtDNAcn. Thus, the moderate reduction of mtDNAcn observed in this study may not have a functional effect on the respiratory chain capacity. Finally, when we integrated the detailed pathologic and cognitive measures with the mitochondrial measures available for our DLPFC samples to disentangle their relationship, we found that both, mtDNAcn and mitochondrial content, were associated with cognitive function and neuronal loss indicating their involvement in neurodegeneration and the need for future studies to interrogate several mitochondrial measures to fully capture mitochondrial health.

## Conclusions

We profiled mtDNA quantity and quality across multiple regions in the aged human brain to study their relation with various age-related pathologies and cognitive function. Overall, lower mtDNAcn was found in regions affected by pathologies, and the loss of mtDNA was primarily related to tau and not to amyloid-β pathology. The large effect of TDP-43 on mtDNAcn in the PCC points to a vulnerability of this region in LATE-NC. Our data further showed that the lower mtDNAcn is not just a consequence of neuronal loss, but directly related to worse cognitive function, and, thus, therapeutics enhancing mitochondrial function could potentially improve cognitive function. In the cortex, the frequency of heteroplasmic mtDNA point mutations and small indels increased with age, but there was little evidence pointing to an association with pathologies, suggesting that small heteroplasmic mtDNA mutations are probably not involved in the pathogenesis of age-related neurodegenerative diseases.

## Supplementary Information

**Additional file 1. Table S1.** Characteristics of the Mayo cohort. **Table S2.** Characteristics of the MSBB cohort. **Table S3.** Detailed results from the univariate regression analyses shown in Fig. 2A. **Table S4.** Association between cell type proportions and mtDNAcn in the DLPFC. **Table S5.** Association between number of mtDNA heteroplasmic mutations and age adjusted for pathologic diagnosis. **Table S6.** Datasets and analysis results deposited at the AD Knowledge Portal. **Fig. S1.** Log-transformed mtDNAcn values are approximately normally distributed. **Fig. S2.** Changes of the mtDNAcn are primarily associated with tau in the DLPFC. **Fig. S3.** Significant fraction of the *APOE* ε4 effect on mtDNAcn is mediated via AD pathologies. **Fig. S4.** Number of mtDNA heteroplasmic mutations in cortical regions is associated with age adjusted for sex, pathologic diagnosis, and mtDNAcn. **Fig. S5.** Mitochondrial content score and its relation to mtDNAcn, mtDNA heteroplasmy burden and AD-related phenotypes.

**Additional file 2.** Supplementary Excel File 1 contains DNA sequencing quality metrics, estimated raw and normalized mtDNAcn, estimated heteroplasmy levels, and phenotype information for all samples from the ROSMAP, Mayo Clinic, and MSBB studies.

**Additional file 3.** Supplementary Excel File 2 contains detailed results from the analysis of the 81 candidate SNPs and their effects on mtDNAcn.

**Additional file 4.** Supplementary Excel File 3 lists the proteins that were used to derive the protein scores for the respiratory chain complexes.

## Data Availability

Raw and processed data (WGS, proteomics, RNA-seq), called variants, and analysis output (mtDNAcn, mtDNA heteroplasmy levels) from the three studies (see Table S6 for details) are available via the AD Knowledge Portal (https://adknowledgeportal.org). The AD Knowledge Portal is a platform for accessing data, analyses, and tools generated by the Accelerating Medicines Partnership (AMP-AD) Target Discovery Program and other National Institute on Aging (NIA)-supported programs to enable open-science practices and accelerate translational learning. The data, analyses and tools are shared early in the research cycle without a publication embargo on secondary use. Data is available for general research use according to the following requirements for data access and data attribution (https://adknowledgeportal.org/DataAccess/Instructions). For access to content described in this manuscript see: 10.7303/syn25618990. Pathologic and phenotypic data from ROSMAP are available on our Resource Sharing Hub at https://www.radc.rush.edu. R and shell scripts used to generate figures and analysis results are deposited at GitHub: https://github.com/cu-ctcn/mtDNA.

## Abbreviations

AD: Alzheimer’s disease
CB: Cerebellum
DLPFC: Dorsolateral prefrontal cortex
FP: Frontal pole
GWAS: Genome-wide association study
LATE-NC: limbic-predominant age-related TDP-43 encephalopathy neuropathological change
MSBB: Mount Sinai Brain Bank
mtDNA: Mitochondrial DNA
mtDNAcn: Mitochondrial DNA copy number
NUMT: Nuclear mitochondrial DNA
PCC: Posterior cingulate cortex
PD: Parkinson’s disease
PSP: Progressive supranuclear palsy
ROSMAP: Religious Orders Study and Rush Memory and Aging Project
SNP: Single nucleotide polymorphism
TCX: Temporal cortex
TMT: Tandem mass tag
WGS: Whole-genome sequencing
